# Hybrid quantum neural networks show strongly reduced need for free parameters in entity matching

**DOI:** 10.1038/s41598-025-88177-z

**Published:** 2025-02-05

**Authors:** Lukas Bischof, Stefan Teodoropol, Rudolf M. Füchslin, Kurt Stockinger

**Affiliations:** 1 Zurich University of Applied Sciences, Winterthur, Switzerland; 2 European Centre for Living Technology, Venice, Italy

**Keywords:** Quantum computing, Entity matching, Experimental evaluation, Quantum information, Computer science

## Abstract

Modern technology and scientific experiments increasingly generate larger and larger amounts of data. This data is sometimes redundant, incomplete or inaccurate and needs to be cleaned and merged with other data before becoming useful for scientific exploration. Hence, entity matching, i.e. the process of linking data about a given entity gathered from multiple data sets, is a major problem in artificial intelligence with applications in science and industry. Typical methods for entity matching either use specialized algorithms or supervised machine learning. Although the problem has been well studied on classical computers, it is unclear how quantum approaches would tackle these challenges. In this paper, we evaluate quantum machine learning algorithms for entity matching on a hand-crafted data set and compare them to similar classical algorithms. We do this by implementing a neural network with a classical embedding layer and extending it with quantum layers. Our experimental results suggest that our hybrid quantum neural network reaches similar performance as classical approaches while requiring an order of magnitude fewer parameters than its classical counterpart. Furthermore, we also show that a model trained on a quantum simulator is portable and thus transferable to a real quantum computer. From a practical perspective and as long as quantum hardware is a scarce resource, experiments, e.g. addressing performance, can profit from producing good initial configurations for quantum neural networks via a simulator, thus only leaving the fine-tuning to quantum computations.

## Introduction

Entity matching, also known as data matching, entity resolution or record linkage, is the process of identifying and matching records that refer to the same entity^1^. Entity matching is a widely used practice when merging datasets from different companies or scientific experiments. In these cases, two different companies might have datasets with duplicate records that needs to be removed before integrating into one joint dataset. Thereby, entities range from address strings, where different address formats may denote the same real-world address, to much more intricate cases such as texts, where different sentences may have exactly or at least in their, with respect to a domain, relevant essence the same meaning. The importance of entity matching can be seen in various domains, such as e-commerce, health services and media management, where it is used to ensure data integrity. In short, entity matching is an important problem in database research, natural language processing or artificial intelligence in general.

Errors during data processing can cause related data to be lost or incorrect. Such kinds of data processing errors are common whenever manual data entry is present or whenever data can get corrupted (i.e., when stored long-term without redundant backups, when transferred over physical media without checksums, scrambled due to electromagnetic interference, etc.). Hence, data cleaning^2^ is a crucial data quality task in data management^3,4^.

Consider, for example, two department stores that collect their product data in separate databases according to their individual standards. These two stores are interested in integrating their business operations, which requires the merging of their product data. Therefore, they must identify which products are identical in both databases. An example excerpt of what this could look like is shown in Table 1.

**Table 1:**
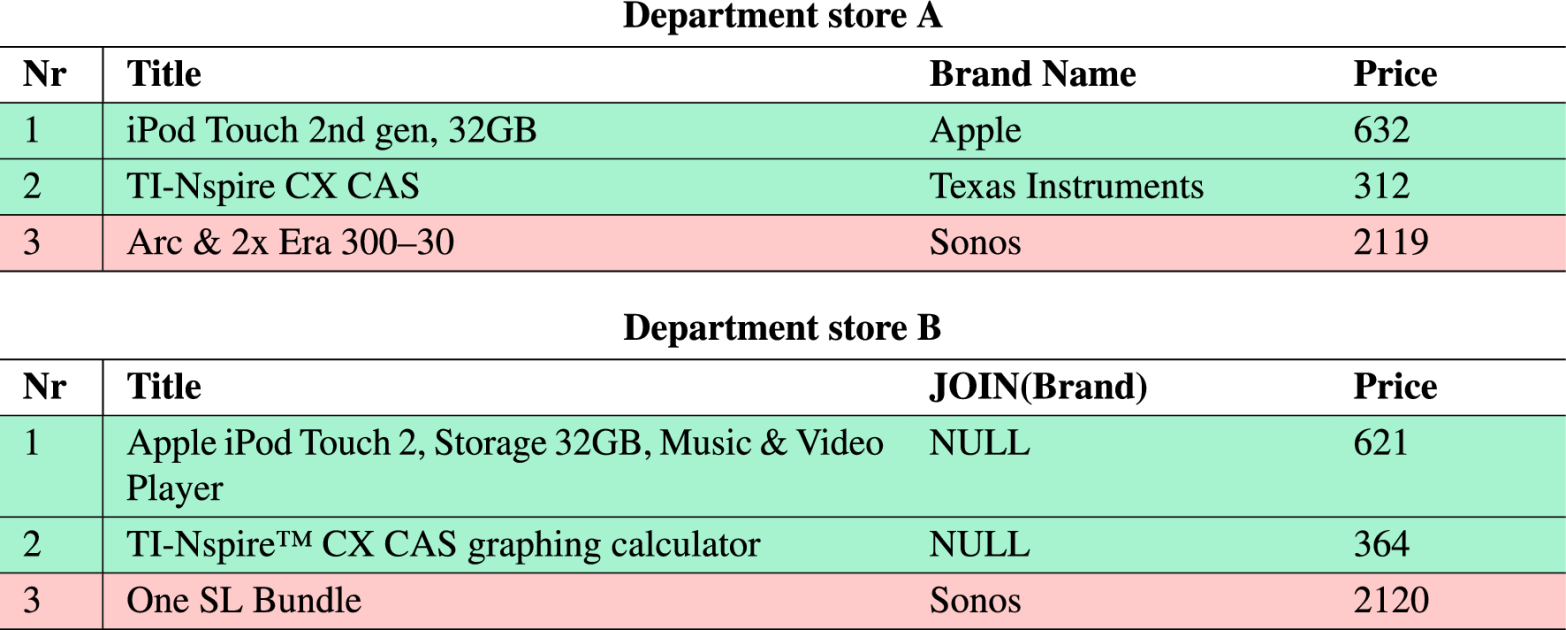
Example excerpt of two fictional department stores’ product data. Green cells indicate the same real-world product listed in both stores while red cells indicate different products. Specifically, the products with numbers 1 and 2 refer to the identical real-world products and are considered as matches. However, they are listed with different titles, brand names and prices which makes it non-trivial to automatically classify them as matches.

In general, entity matching is easy if distance measures in the space of records map (at least in most cases) monotonically to a distance measure between entities. In many practical cases, the situation is much more involved. First, proximity with respect to a measure in record space does not translate into proximity of entities and vice versa (similar records may have different meanings and entities with the same meaning may be represented by completely different records). And, second, explicit distance measures may not be known; in such cases, one often produces an implicit measure by some form of learning.

Consequently, and again referring to the example above, the companies need a method to detect and merge these duplicate documents into one real-world entity. These methods must be capable of tolerating various types of errors such as misspellings and they must treat different formats, different levels of detail, and different languages, etc. properly.

To tackle this problem, the use of machine learning seems promising when considering the recent advancements in the field^5,6^. Implementations of entity resolution and entity matching systems already exist based on pre-trained language models as well as recurrent neural networks (RNNs) and word embeddings^7,8^ or transformer-based approaches^9–11^.

Despite the advancements in the field, entity matching remains a challenging task—basically because standardization is either not wide-spread or the data is not formalized. In the past few years, quantum computing and, with it, quantum machine learning has become more popular^12–15^ and accessible to the public. Especially the release of Python libraries such as Qiskit^16^ and Cirq^17^ for development and testing accompanied by the release of publicly available quantum computers contributed to further progress in the field. In this article, we show that quantum machine learning is a promising approach for the problem of entity matching. In particular, we demonstrate that the quantum analogue of a neural network requires much fewer parameters than its classical counterpart. Quantum computers are not yet widely available. Performing research and testing algorithms is therefore expensive. Technically relevant is our finding that simulators are well capable of providing testbeds for studies of quantum circuits and their behavior not only for the well-known algorithms but also for the more general problem of entity matching.

In this paper, we will study the feasibility of using quantum machine learning to tackle the entity matching problem. The contributions of our paper are as follows.

1. We *construct a dataset* that is small enough to be used for entity matching on an openly available quantum computer with a reasonable amount of qubits. (During the writing of this paper, quantum computers with up to 127 qubits were available on IBM’s quantum cloud.)
2. We implement a classical embedding layer that takes the entities to be matched and converts them to a fixed-size embedding vector. By extending the embedding vector with a quantum layer, we introduce a *quantum classifier*. We train two machine learning models, one using an optimizer that considers both the classical and quantum part of the neural network (*hybrid quantum neural network*), and one that only considers the quantum part isolated (*quantum neural network*).
3. We compare the performance of the quantum classifiers to a classical classifier using the term frequency-inverse document frequency (TF-IDF)^18^ as well as to classical neural networks. This method has been chosen because we aim at comparing the number of parameters needed in quantum and classical approaches while achieving comparable performances. TF-IDF is an established and well-understood method with limited parameter requirements. We could use more advanced methods, e.g. transformer-based approaches, which perform better than TF-IDF but at the price of largely increased number of parameters, which would skew the intended comparison.
4. Our experimental results on real quantum computers show that the *hybrid quantum neural network at least matches the performance of a classical counterpart while reducing the number of parameters*. Furthermore, we demonstrate that a machine learning model trained on a quantum simulator can be transferred to a real quantum computer with comparable results. We used entity matching as a non-tivial test problem because many practical questions can be interpreted in its context and setting.

To the best of our knowledge, this is the *first work to tackle the entity matching problem using quantum machine learning*.

## Fundamentals of quantum computing and quantum machine learning

In this section we introduce the fundamentals of quantum computing and quantum machine learning. The reader familiar with these concepts can directly continue with “Approach: quantum neural networks for entity matching”.

### Quantum computing

For an in-depth understanding of quantum computing and the nomenclature used in this paper, we refer the reader to the literature^19^. However, to provide a self-contained paper, we will briefly introduce the basic concepts of quantum computing and then dive deeper into the aspects of quantum machine learning that are relevant to this paper.

At the core of quantum computing lies the qubit, the quantum analogue of the classical bit that acts as the fundamental information unit. Unlike classical bits, qubits are associated with a two-state property of a physical system (e.g., the spin of an electron) and are described by a state vector in a two-dimensional complex vector Hilbert space.

With $\left| 0\right\rangle$ or $\left| 1\right\rangle$ as orthonormal base vectors of this Hilbert space, a general qubit $\left| \psi \right\rangle$ is given by $\left| \psi \right\rangle = \alpha \left| 0\right\rangle + \beta \left| 1\right\rangle$ where $\alpha$ and $\beta$ are complex numbers satisfying the normalization condition $|\alpha |^2 + |\beta |^2 = 1$. In contrast to conventional bits, a qubit cannot be read out completely, which means that there is (in general) no way to determine the values $\alpha , \beta$ of a single qubit. But a qubit can be measured with respect to a given base. A measurement described by, say, $\left| 0\right\rangle$ transforms the system given by $\left| \psi \right\rangle$ into the state represented by $\left| 0\right\rangle$ with probability $|\alpha |^2$ and accordingly $\left| 1\right\rangle$ with probability $1 - |\alpha |^2 = |\beta |^2$.

Qubits can be combined into quantum registers. The joint space of *n* qubits is the tensor product of the individual qubit spaces: $\mathscr {H} = \mathscr {H}_1 \otimes \mathscr {H}_2 \otimes \ldots \otimes \mathscr {H}_n$ with a dimension of $2^n$. For instance, the two independent qubits $\left| \psi _1\right\rangle = \alpha _1\left| 0\right\rangle + \beta _1\left| 1\right\rangle$ and $\left| \psi _2\right\rangle = \alpha _2\left| 0\right\rangle + \beta _2\left| 1\right\rangle$ form the joint state $\left| \psi \right\rangle = \left| \psi _1\right\rangle \otimes \left| \psi _2\right\rangle = \alpha _1\alpha _2\left| 00\right\rangle + \alpha _1\beta _2\left| 01\right\rangle + \beta _1\alpha _2\left| 10\right\rangle + \beta _1\beta _2\left| 11\right\rangle$. For notational convenience, if one applies the same operator *U* on each qubit of a quantum register, one writes $U\otimes ... \otimes U = U^{\otimes n}$ and $\left| 0\right\rangle \otimes ... \otimes \left| 0\right\rangle = \left| 0\right\rangle ^{\otimes n}$

In general, the evolution of a quantum system is described by a unitary transformation acting on the full state space (A unitary transformation can be represented by a matrix *U* that satisfies the condition $U^\dagger U = I = U U^\dagger$, where $U^\dagger$ is the conjugate transpose of *U* and *I* is the identity matrix.). This means that the state of a quantum register $\left| \Psi \right\rangle$ can be transformed by applying a unitary transformation *U* to it:

1 $$\begin{aligned} \left| \Psi '\right\rangle = U\left| \Psi \right\rangle \end{aligned}$$

**Fig. 1:**
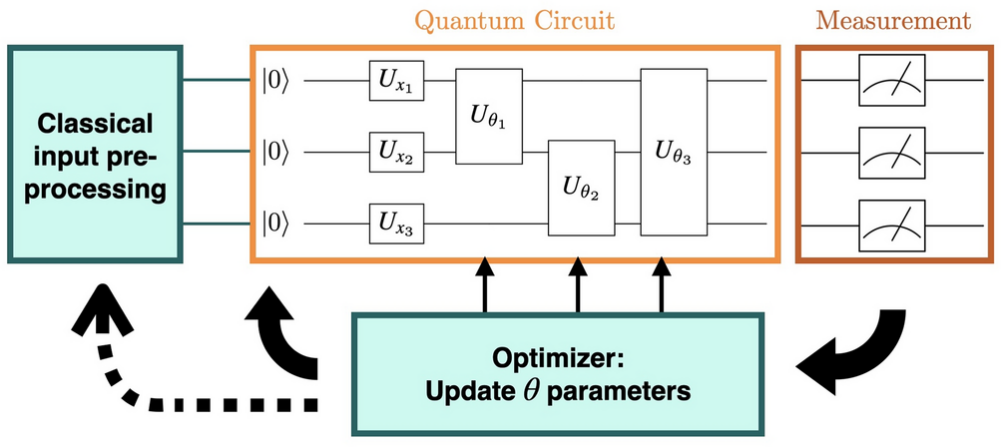
The principle of learning using variational quantum circuits. Some quantum circuit $U(\vec {x}, \vec {\theta })$ is parameterized by $\vec {\theta }$ and a classical input $\vec {x}$, that has previously been prepared by some classical preprocessor. Based on the measurement outcome, the parameters are updated using a classical optimization algorithm. The optimizer can also be linked to the classical preprocessor to optimize the state preparation. This process is repeated until the cost function converges to a minimum.

In general, *U* is a function of time $U=U(t)$ and derived from the Schrödinger equation. Gate-based quantum computing is characterized by a sequence of such unitary transformations (so-called quantum gates), as depicted in the quantum circuit part of Figure 1. Two remarks: First, the quantum gates may affect only a subset of the qubits building up the quantum register. Second, in contrast to the general case, the individual quantum gates are assumed to be time-independent but can be parameterized.

### Quantum machine learning

Machine learning (ML) and especially neural networks (NN) have proven themselves to be a powerful tool for solving complex problems in a variety of domains by automating solutions to problems by learning from data^20^. ML differs from the traditional approach to programming, where the programmer has to explicitly define rules used to solve a problem.

With the rise of quantum-enhanced processing, researchers began to explore the potential of quantum computing for machine learning (QML)^21^. It has already been shown that QML can be used to solve problems at least as accurately as classical ML algorithms or even outperform them in certain tasks^15,22^.

#### Learning the semantics of entities using quantum computers

Enabling a quantum computer to learn semantics, i.e., the relationship between records and entities, is a challenging task for at least two reasons.

Firstly, one needs a way to feed the quantum process with data. Unlike classical computers, quantum computers are not able to read data from a source directly and we currently lack the tools that would help us do such as QRAM^23^. This is mostly because of a fundamental fact, the so-called no-cloning theorem, which rules out the existence of a simple copy-and-paste process for physical reasons; e.g., the possibility of copying quantum registers would provide a mean to break the uncertainty principle. One needs an efficient way to encode the data into an input quantum state, each time one runs a quantum computation.

Secondly, the quantum process needs to be able to learn from the data. To achieve this, we have to interpret the measured results of the quantum process in a meaningful way and use them to either update the “input” quantum state or the parameters of the quantum process. However, since we are only able to read the output in a probabilistic way, optimizing the parameters of the quantum process may require a large number of iterations.

#### Quantum neural networks

In order to imitate the behavior of artificial neurons used in classical neural networks on a quantum computer, we employ parameterized quantum gates. The parameters are summarized in a vector $\vec {\theta }$; a parameter $\theta _i$ can be understood as describing a rotation around some axis realized by its according unitary operator in the $2^n$ dimensional state space.

The according circuit paradigm is called a *variational quantum circuit* (VQC)^24,25^, also known as *parameterized quantum circuit*.

VQCs consist of three parts^26^:

1. An initial *n*-qubit input state, which usually is a zero-state $|0\rangle ^{\otimes n}$. Any other device preparing a special input state would also be conceivable .
2. A quantum circuit $U(\vec {\theta })$ consisting of unitary transformations parameterized by $\vec {\theta }$, a vector of free parameters.
3. The measurement of the observable $\hat{O}$:
  2 $$\begin{aligned} f(x,\vec {\theta }) = \langle \hat{O} \rangle = \langle 0 | U^\dagger (x, \vec {\theta }) \hat{O} U(x, \vec {\theta }) | 0 \rangle \end{aligned}$$

Based on a VQE, one realizes a hybrid quantum-classical approach according to^27^ as illustrated in Fig. 1. Thereby, after the quantum computation, the parameter vector $\vec {\theta }$ is updated using classical means and the process is iterated.

Usually, in quantum neural networks, the quantum circuit itself is composed of three steps^28^:

First, a feature map *F* that maps a a vector of classical data points $\vec {x}$ to an *n*-qubit quantum state $\left| \varphi \right\rangle ^{\otimes n}$^14^:

3 $$\begin{aligned} \left| \varphi (\vec {x})\right\rangle = F(\vec {x}) \left| 0\right\rangle \end{aligned}$$

Second, an ansatz *A* (the analog of the classical neural net) that operates on the prepared quantum state $\left| \varphi (\vec {x})\right\rangle$ using gates parameterized by $\vec {\theta }$:

4 $$\begin{aligned} \left| \psi (\vec {x}, \vec {\theta })\right\rangle = A(\vec {\theta }) \left| \varphi (\vec {x})\right\rangle \end{aligned}$$

Finally, an observable $\hat{O}$ that is measured on the output state $\left| \psi (\vec {x}, \vec {\theta })\right\rangle$, recording one of the eigenvalues of $\hat{O}$ as the prediction (analogous to Eq. 2):

5 $$\begin{aligned} \langle \hat{O} \rangle = \left\langle \psi (\vec {x}, \vec {\theta })\right| \hat{O} \left| \psi (\vec {x}, \vec {\theta })\right\rangle \end{aligned}$$

**Fig. 2:**
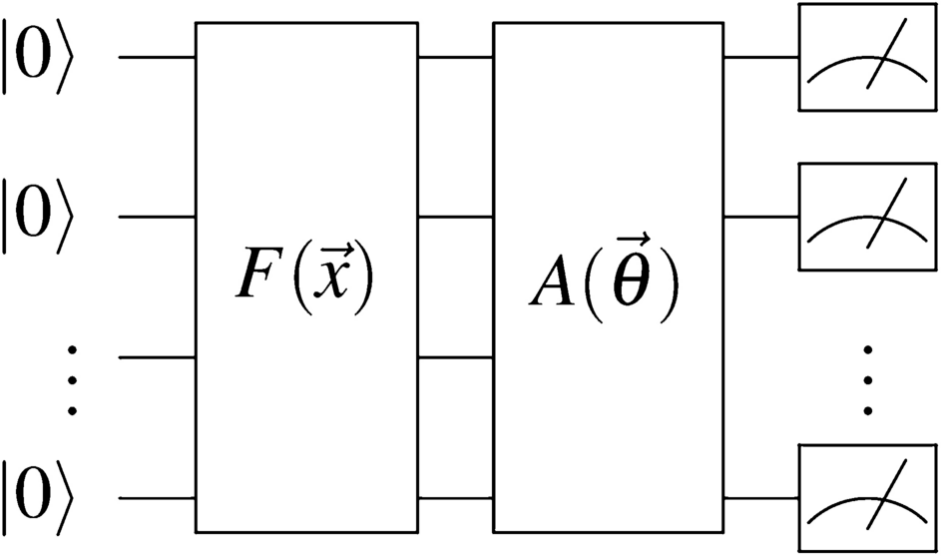
The schema of a quantum neural network. The feature map *F* maps the classical data point *x* to a quantum state $\left| \varphi \right\rangle$. The ansatz *A* is a parameterized quantum circuit that operates on the quantum state $\left| \varphi \right\rangle$. The observable $\hat{O}$ is measured on the output state $\left| \psi \right\rangle$.^28^.

The resulting structure of the quantum circuit is shown in Fig. 2:

Generally, the classical data should be encoded in a way that the feature value can be easily extracted from the quantum state^28^. Over the years, several ways of encoding classical data into quantum states have been proposed^29,30^, such as basis encoding, angle encoding, amplitude encoding, ZFeatureMap, ZZFeatureMap, or any arbitrary encoding.

The ansatz is the part of the quantum circuit that is responsible for the actual computation; presently, several possibilities are subject to intensive scrutiny, see e.g.^15,31,32^. Finally, the measurement of the observable has to be used to optimize the parameters in order to achieve the best possible performance, which is done by using a classical optimization algorithm.

To reduce computational costs, there are other, approximation methods to compute the gradients of the sampling probabilities such as the Simultaneous Perturbation Stochastic Approximation (SPSA)^33^ algorithm that only requires two circuit evaluations regardless of the number of parameters.

## Approach: quantum neural networks for entity matching

We compare two different approaches of quantum neural networks for entity matching. Both approaches use a combination of classical and quantum layers as shown in Fig. 1. However, the training is done differently as we will discuss below.

### Quantum neural network (QNN)

**Fig. 3:**
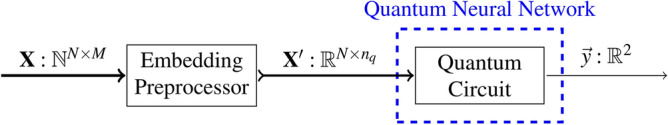
Schematic architecture of the quantum neural network (QNN). The input data $\textbf{X}$ is an $N \times M$ matrix with *N* integer-encoded sentence pairs and a maximum token capacity of *M* per row. Every row represents a sentence pair and is a zero-padded sequence of tokens, representing the individual words. Every sentence pair is transformed by the “Embedding Preprocessor” into a deterministic fixed-size vector representation with dimensionality $n_q$. The transformed matrix is then fed into the quantum circuit. The sampler output of the quantum circuit is used by an interpretation function $\sigma (\vec {y})$ to classify the measured values into a match or non-match.

This approach examines a simple approach to quantum machine learning for entity matching. It uses a standalone quantum neural network for training and prediction. The high-level architecture of the approach is shown in Fig. 3.

We now describe the QNN approach in detail:

1. *Data preparation*: The raw input data (entities) is preprocessed by removing punctuation and converting all characters to lowercase. Furthermore, the data is encoded into a numerical representation by substituting each word with a unique integer.
2. *Embedding preprocessor using classical neural network*: It takes the role of the classical preprocessor for the data, mentioned in Fig. 1. The embedding preprocessor converts the input data into a fixed-size vector representation. The output of the neural network is a single dense layer representing a match or non-match of the two entities. This layer is only used for training but not as a classifier, i.e. for deciding of an entity is a match or not.
3. *Quantum neural network*: The QNN consists of a single quantum layer, which is used to determine if the two provided entities match or not. This is done by applying an interpretation function $\sigma (\vec {y})$ to the sampler output of the quantum layer, specifically a parity function.
  The quantum layer uses a ZZFeatureMap^34^ to encode the input data into a quantum state and a RealAmplitudes module with linear entanglement to classify the data (In linear entanglement qubit *i* is entangled with qubit $i+1$, for all $i \in \{0,1,...,n - 2\}$ where *n* is the total number of qubits.). The RealAmplitudes module applies alternating layers of RY rotations and CNOT gates to produce entanglement.
  Training of the QNN model is done using the Adam optimizer. The learning rate is dynamically adjusted using the ReduceLROnPlateau scheduler of PyTorch.

### Hybrid quantum neural network (HQNN)

The previous approach uses an independent classical neural network as an embedding preprocessor and an independent quantum neural network as a classifier. This approach uses a hybrid quantum neural network where the embedding processor and the classifier are trained jointly. The neural network consists of a classical embedding layer, which acts as the input layer for the quantum layer. The quantum layer is the final layer of the neural network and is used to determine if the two provided entities match or not. Overall, compared to the QNN, the HQNN uses a *dynamic embedding layer*, and a *more complex quantum layer*. The data preparation step is the same as for the QNN.

**Fig. 4:**
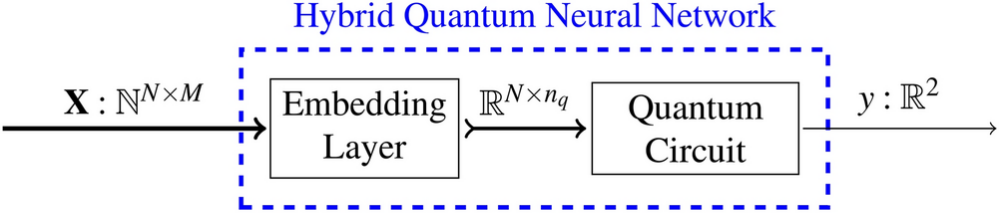
Schematic architecture of the hybrid quantum neural network (HQNN). The input data $\textbf{X}$ is the same as for the HQNN (see Fig. 3): an $N \times M$ matrix with *N* integer-encoded sentence pairs and a maximum token capacity of *M* per row. The input gets fed directly into the neural network, where it is received by an embedding layer. The embedding layer reduces the dimensionality of the input data to $n_q$ dimensions, which is the *number of qubits* of the quantum circuit. The sampler output of the quantum layer is then used by an interpretation function $\sigma (\vec {y})$ to classify the measured values into a match or non-match. The returned vector $\vec {y}$ then denotes the probability of a match and a non-match. The dashed blue box denotes the trainable part of the model that is optimized during training.

The high-level architecture of the HQNN is shown in Fig. 4.

**Fig. 5:**
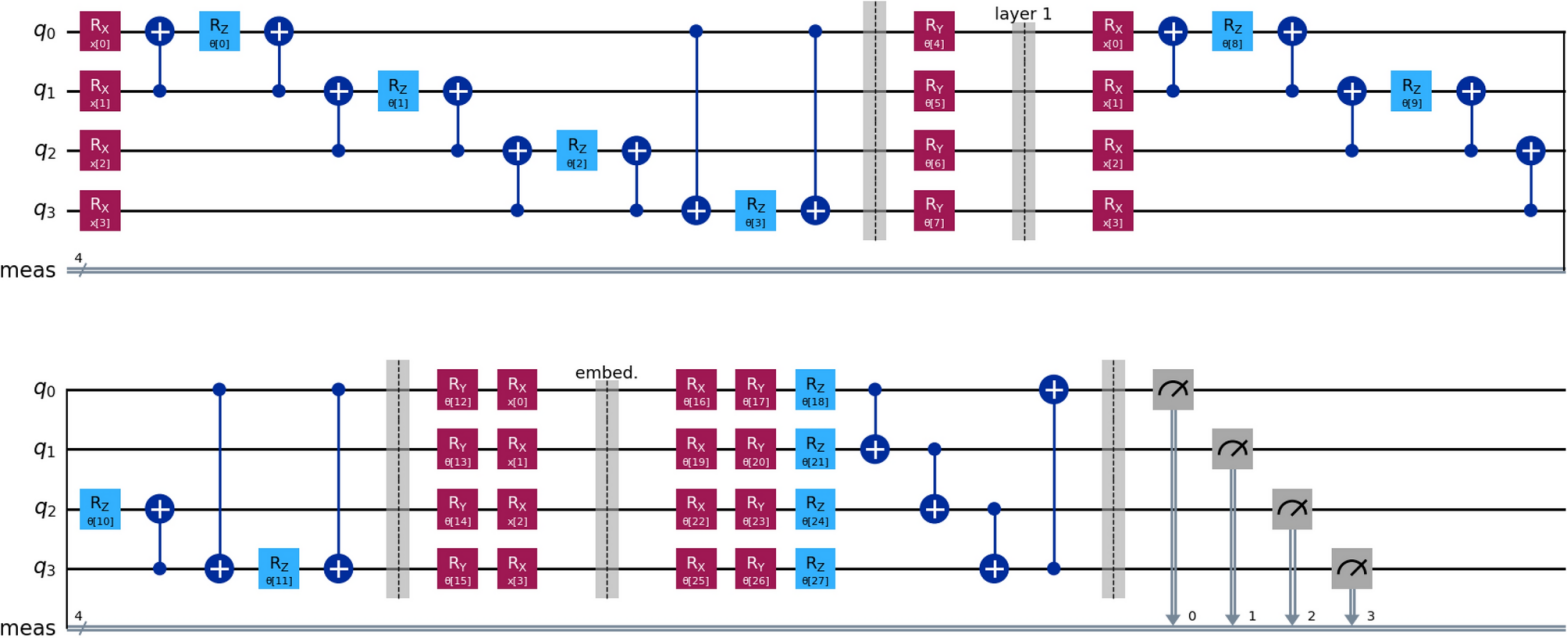
Example quantum circuit of the HQNN model using four qubits. Before the “embed” barrier, the QAOAEmbedding layer encodes the input data into a state vector. In this example, the QAOAEmbedding layer is repeated twice. After the “embed” barrier, an entanglement layer is applied as an ansatz. The entanglement layer is PennyLane’s StronglyEntanglingLayers implementation.

We now describe the HQNN approach in detail:

1. *Embedding Layer*: The embedding layer is part of a hybrid classical quantum neural network which is trained together with the quantum circuit.
  The parameters are optimized during training and are adjusted to the needs of the quantum layer.
2. *Quantum Circuit*: A quantum layer is used as the final layer of the neural network, whose sampler output is used to determine if the two provided entities match or not. The quantum circuit is shown in Fig. 5. It uses Pennylane’s^35^ QAOAEmbedding layer structure to encode the input data into a quantum state and afterward applies an entanglement layer. The entanglement layer is implemented using a Qiskit version of Pennylane’s StronglyEntanglingLayer module.
  The QAOAEmbedding is a feature map inspired by the QAOA ansatz^30^. This layer applies two circuits: The first encodes the features, and the second is a variational ansatz, inspired by the 1-dimensional Ising model^36^. The feature encoding layer transforms classical input data into a quantum state using the angles of a parametrized RX rotation gate. The following Ising model inspired ansatz consists of multiple trainable two-qubits ZZ interactions $e^{-i\frac{\alpha }{2}\sigma _z \otimes \sigma _z}$ and trainable local fields $e^{-i\frac{\beta }{2}\sigma _\mu }$ where $\sigma _\mu$ is a Pauli operator (either $\sigma _x$, $\sigma _y$ or $\sigma _z$) and $\alpha ,\beta$ are trainable parameters. In our implementation, we use the RY gate as the default Pauli operator.
  The entanglement layer inspired by PennyLane’s StronglyEntanglingLayer^37^ module uses a parametrized rotation gate $R(\alpha _q^l, \beta _q^l, \gamma _q^l)$ on each qubit *q* for each layer *l* and a series of CNOT gates to entangle the qubits. The entanglement is done in a linear fashion, meaning that each qubit *q* is entangled with the next qubit $q+1$ in the chain for $q\in \{0,1,\ldots ,n-2\}$, where *n* is the number of qubits.
  Optimization of all parameters (embedding and quantum layer) is done using the Adam optimizer. The learning rate is dynamically adjusted using the ReduceLROnPlateau scheduler of PyTorch.

### Classical approaches

To highlight the benefits of quantum approaches, we compare the quantum models with two classical approaches. We use TF-IDF and cosine similarity^38^ as a classical unsupervised approach for entity matching. This comparison serves as a reasonable benchmark for performance.

Furthermore, we evaluate a classical neural network model with an architecture similar to the quantum neural network model by replacing the quantum layer with an LSTM layer^39^ with properties as follows:

- An embedding layer receiving the input with an output dimension of 16, using 848 trainable parameters.
- A subsequent dropout layer with a dropout rate of 0.3.
- An LSTM layer with 16 hidden units, using 4,224 trainable parameters.
- A global average pooling layer.
- Another dropout layer with a dropout rate of 0.1.
- A dense layer with a single output and sigmoid activation function, using 33 trainable parameters.

The model is trained using the Adam optimizer with a learning rate of 0.01 and binary cross-entropy loss. Subsequently, in this paper, we refer to this model as the *NN with LSTM*. The choice of an NN with LSTM is motivated with the intention to compare the parameter requirements of a classical method with a quantum approach. An NN with LSTM exhibits a performance similar to the best quantum approach we tested and uses in the setting under consideration a couple of thousand parameters. Concerning performance, better classical methods are available but they all reuqire much more parameters (e.g. transformer architectures requiring orders of magnitude more parameters than LSTMs.)

## Experiments and results

The aim of this work is to evaluate the performance of the approaches described in “Approach: quantum neural networks for entity matching” on a simplified dataset described in “Dataset”. The focus of this work is to evaluate the feasibility of current quantum computing technology for a real-world problem. We specifically address the following research questions:

- How do quantum approaches compare to a classical approach in terms of number of required parameters and performance?
- How portable are the quantum approaches to real quantum hardware?
- How well does a simulation of quantum approaches on a classical computer scale to real quantum hardware?

### Dataset

Entity matching is a problem not restricted to a specific data type. However, in this paper, we will work with a synthetic dataset using single, short sentences, mainly due to the limited amount of quantum hardware available. Constructing a dataset for benchmarking entity matching problems is common practice, and can be done by modifying existing datasets, as done in^11^, or by creating a new dataset from scratch, which is specifically useful for quantum-enhanced NLP tasks, see e.g.^40^. We extend the approach of^40^ by hand-crafting a dataset that is specifically designed for entity matching. By hand-crafting the dataset, we can ensure that the problem is small enough to be solved on current quantum hardware, but still complex enough to be interesting. This means that, in our case, the documents consist of a variable number of words that form one sentence with up to ten words. These sample sentences refer to a number of different meanings (entities). To make it an interesting classification problem, different arrangements of words refer to a similar meaning (e.g., “Many trees grow in a forest.” and “A forest is a place with trees”).

Our dataset design is a balance between two extreme cases: (1) Entity matching of sets of words without grammar and (2) fully grammatically correct sentences. Extreme case (1) challenges the entity matching algorithm in identifying similar words, subwords, etc. while (2) enables the algorithm to learn matches based on sentence structure. Given a limited number of qubits and thus a limited number of words we can use for our entity matching problem, we have chosen a mixture of short sentences with and without grammatical structure to study the extreme cases of our algorithm.

#### Structure of the dataset

The resulting dataset consists of sentence pairs with labels indicating whether two sentences refer to the same entity or not. Each sentence is created using a dictionary containing 52 words. Each sentence is no longer than 10 words and no shorter than 3 words and is fairly simple in terms of grammar and word choice.

To construct the data, we manually wrote sentences which fall into one of three greater topics, namely forest/nature, cities, and Romeo and Juliet, and grouped them into matching pairs within the respective topics. Afterward, using a simple selection algorithm, we created non-matching pairs using the following rules:

- Randomly select two sentences from a different topic.
- Randomly select two sentences within the same topic but with different meanings. Note that in addition to automatically verifying the meaning, i.e. the similarity, between two entities, we also evaluated the similarities of the test set by humans.
- Generate a sentence by randomly selecting words from the dictionary.

Using the resulting list of matching and non-matching pairs, we enhanced the dataset using a mixture of the following rules:

- Mirror the sentences in a pair: Swap the left and right sentence in a pair, retaining the label.
- Create easy-to-detect matching pairs where the sentences are identical.

The generated dataset consists of 2,000 training pairs and 492 testing pairs, resulting in a train/test split of approximately 80%/20% with 75% of the pairs being labeled as non-matching and 25% as matching. By balancing the rules used to create the dataset, we ensured that the majority of the pairs are non-matching, reflecting a more realistic scenario, as in a real-world application, when comparing each list entry with all other entries, matches are less common. Furthermore, it allows us to create a wide range of different pair types, ranging from trivial mismatches to more complex and harder-to-detect sentence pairs. Some examples of the resulting dataset are shown in Table 2.

**Table 2:** Examples of sentence pairs in the dataset. A label of 1 indicates that the two sentences are a match, while a label of 0 indicates that they are not. The sentence pairs reflect a variety of style and grammatical differences, to provide a foundation for the model to generalize meaning.

#	Sentence 1	Sentence 2	Label
1	Romeo dies for Juliet	Romeo died for Juliet	1
2	In a forest a tree dies	In the forest a tree dies	1
3	A tree typically does not grow in a city	Typically a tree does not grow in a city	1
4	People live in cities	People are living in cities	1
5	Houses buildings with place	Are in the city	0
6	Juliet lives in house	The city does not have trees	0
7	A city is a place with many buildings	The forest is a place with trees	0

For a high-level understanding of the dataset, we provide a histogram of the similarity scores of each pair in the dataset. The similarity score is a measure of how close two sentences are, and is determined by comparing word vectors (multidimensional representations of words) of the two sentences. The word vectors and similarity scores are calculated using pre-trained models from the SpaCy library^41^, a popular natural language processing library.

**Fig. 6:**
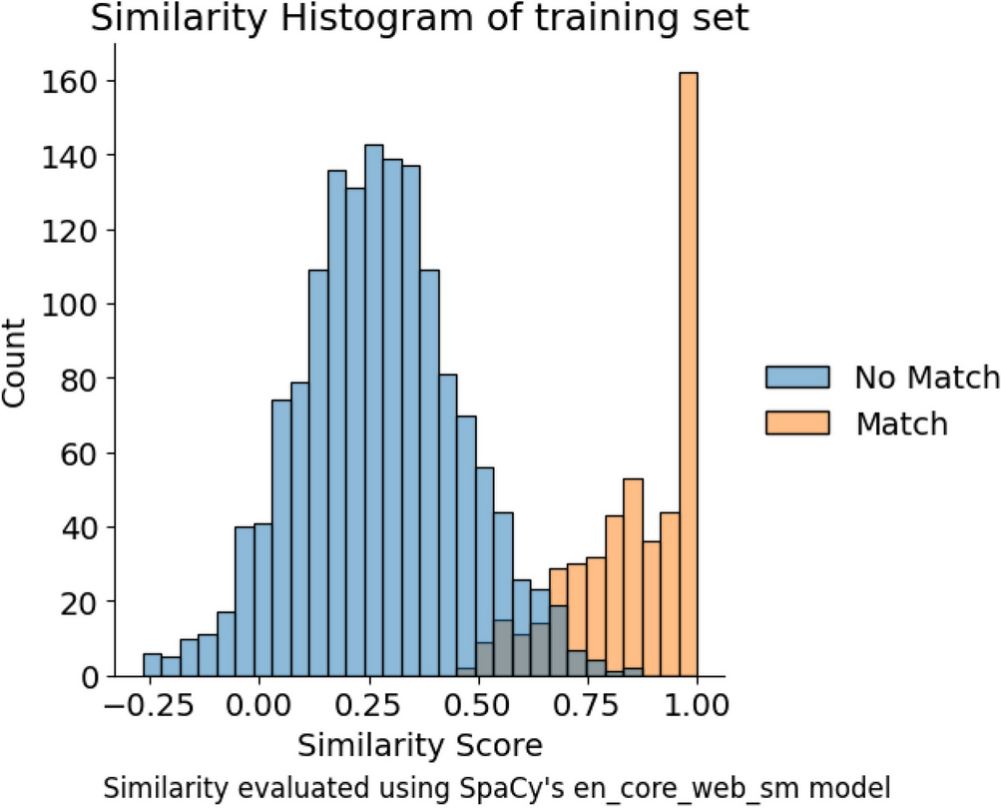
Histogram of the similarity scores of each sentence pair in the training dataset. The x-axis shows the score of SpaCy’s similarity function, while the y-axis shows the number of pairs. The test set shows a similar distribution.

The histogram in Fig. 6 shows us the following:

- Many pairs have a similarity score of 1 or almost 1, indicating that the sentences are almost identical. Classifying these pairs is classically trivial and shows baseline performance if the model is able to classify them correctly.
- The majority of the pairs have a similarity score of zero to 0.3, indicating that the sentences are dissimilar. Classifying most of these pairs is also trivial using classical vector similarity measures.
- The range of similarity scores between 0.3 and 0.8 is the most interesting, as it contains pairs that are hard to classify and cannot be easily separated using classical methods. A good performance in this range would indicate that the quantum model is able to classify pairs beyond the capabilities of traditional vector-based methods.

### Performance results

We evaluate all algorithms using the metrics accuracy, precision, recall and F1-score. For all algorithms, we also report the number of trainable parameters as an indicator for the complexity of the respective approaches.

The training of the models was done using the Qiskit Aer simulator. After training, the models were evaluated on the 27-qubit IBM Hanoi quantum computer as well as on the simulator. Additionally, we trained the HQNN model on the IBM quantum computer to compare the results with the simulator-trained models.

The results are summarized in Table 3 showing the quantum approaches at the top and the two classical approaches at the bottom (see last two rows). The quantum neural networks outperform their classical counterparts in terms of accuracy and F1-score, more importantly, they do so with one order of magnitude fewer parameters than the neural network with an LSTM layer. We note that the (classical) TF-IDF approach outperforms all other approaches in terms of accuracy and F1-score.

**Table 3:**
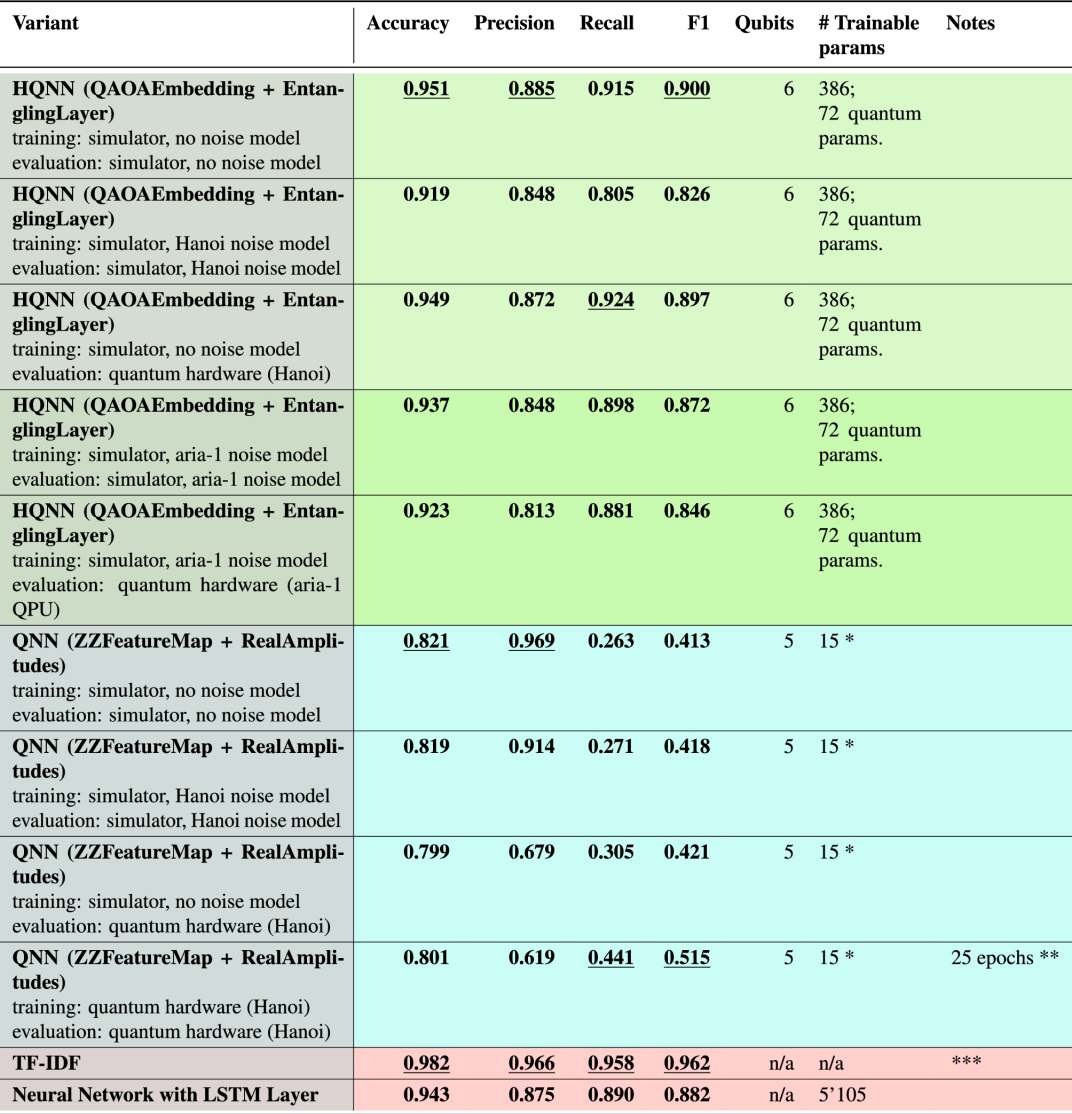
Results of the models tested. The rows are grouped by model, with blue indicating the QNN model, green indicating the HQNN model, and red indicating the classical models. The best result for each metric and within each model is underlined. The name Hanoi refers to the IBM “Hanoi” quantum computer, a 27-qubit system. * Ignores the parameters used for generating the embedding vector before the QNN training started. ** Model started with a “head start” of 5 epochs of classically simulated training. *** TF-IDF threshold was found by stepping though the values from 0 to 1 in steps of 0.01 and then checking for the best accuracy score on the training set.

#### Quantum neural network

The results in Table 3 show that the QNN with ZZFeatureMap + RealAmplitudes has the lowest accuracy and F1 score of all quantum approaches. These results are expected since the QNN only has a limited number of freely available parameters.

Nevertheless, it proved to be a good starting point for more complex models as it was clearly able to learn from the dataset. Interestingly, this model started to perform better when trained on the real quantum computer. The training was done by giving the model a bit of a “head start” by training the first epochs on the simulator and then continuing to train the model on the quantum computer.

#### Hybrid quantum neural network

The HQNN model outperforms the QNN model in all metrics and provides a stable prediction, even on the real quantum computer (see first 5 rows of Table 3).

**Fig. 7:**
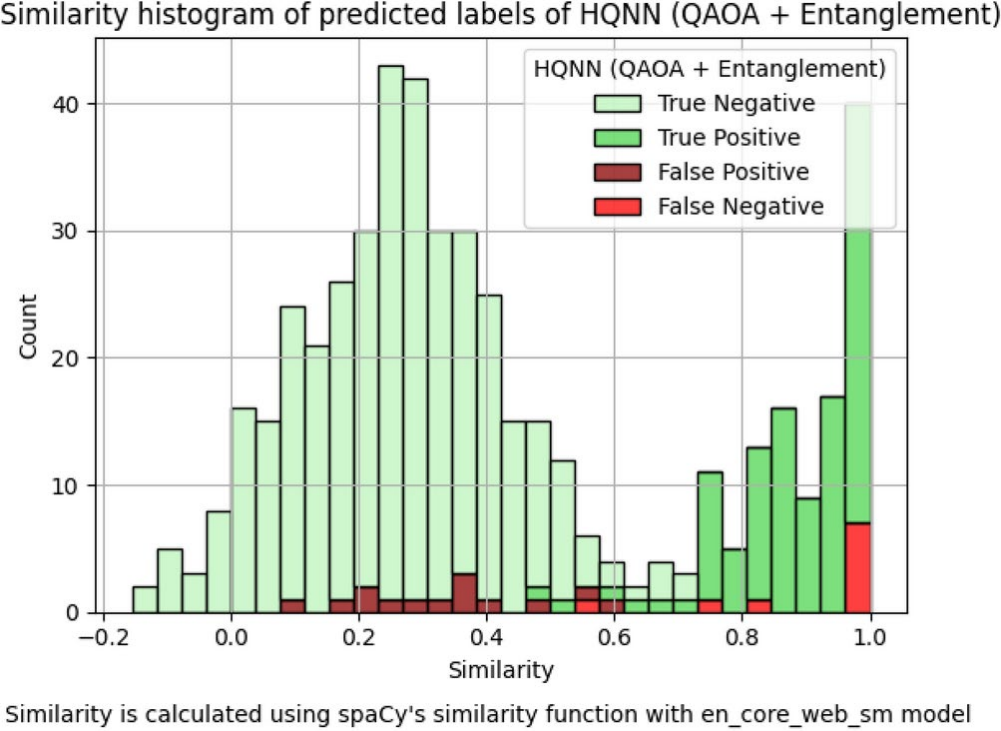
Histogram of the HQNN predictions by the similarity scores of each sentence pair in the test set. The x-axis shows the score of SpaCy’s similarity function, while the y-axis shows the number of pairs. The color of the bars indicates the prediction of the model.

**Fig. 8:**
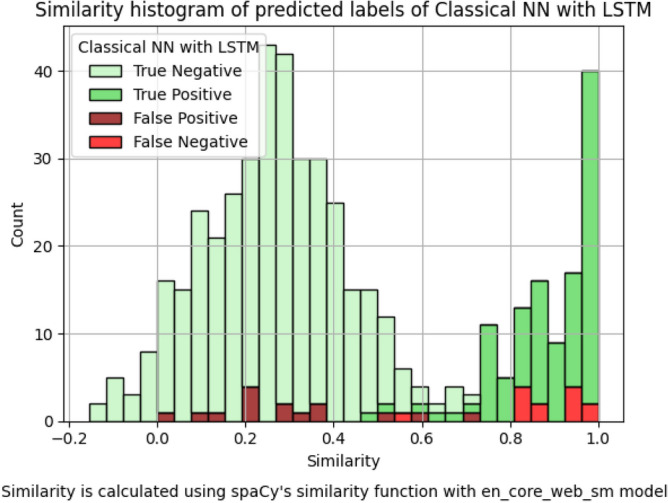
Histogram of the predictions of the NN with an LSTM model by the similarity scores of each sentence pair in the test set. The x-axis shows the score of SpaCy’s similarity function, while the y-axis shows the number of pairs. The color of the bars indicates the prediction of the model.

A closer look at the results reveals that the model has difficulties with sentence pairs that are identical and seldom with pairs that are very different (see Fig. 7). We analyzed the prediction outcomes of the model for the test set sorted by the similarity of the sentence pairs. Note that this behavior is also observed in the classical NN with an LSTM model, as shown in Fig. 8, but there it is not as concentrated on the extremes. Ultimately, these types of errors can be filtered out easily by doing a byte-wise comparison, or by using a threshold-based classical approach like TF-IDF for pre-filtering.

We also evaluated the model on IonQ’s QPU by first simulating it on their dedicated simulator and then running it on the QPU. We see a similar performance with the quantum simulator as on the real quantum computer, even though they use a different architecture. Also, the performance of the purely simulated model is comparable to the one on the real quantum computer, which is a good sign for the model’s generalization and ability to be transferred to different quantum computers.

#### Term frequency-inverse document frequency

The classical TF-IDF approach outperforms all other models in all metrics. Given the rather simple nature of the dataset, this is not surprising. TF-IDF especially performs well in the extreme cases where the sentence pairs are either very similar or very different. Difficulties arise when the pair of sentences are very similar, but have a completely different meaning. To overcome this issue, a combination of the TF-IDF approach with the quantum models could be beneficial, in particular as a pre-filtering step.

### Combination of quantum and classical approaches

**Table 4:**
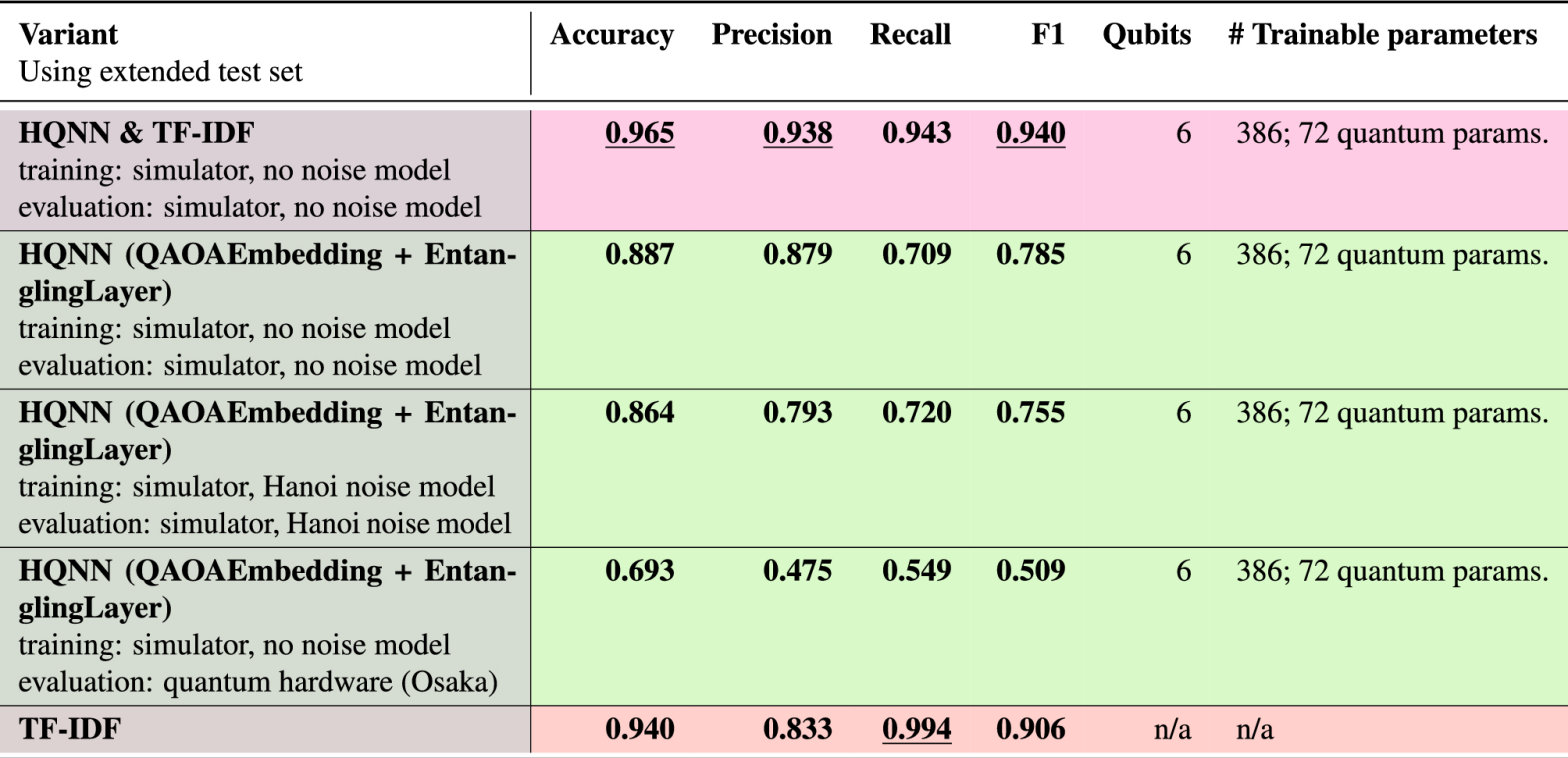
Results of the models tested on the extended dataset. The rows are grouped by model, with pink indicating the combined model, green indicating the HQNN model, and red indicating the classical models. The name Hanoi refers to the IBM “Hanoi” quantum computer, a 27-qubit system, and Osaka refers to the IBM “Osaka” quantum computer, a 127-qubit system. The best results are underlined.

**Fig. 9:**
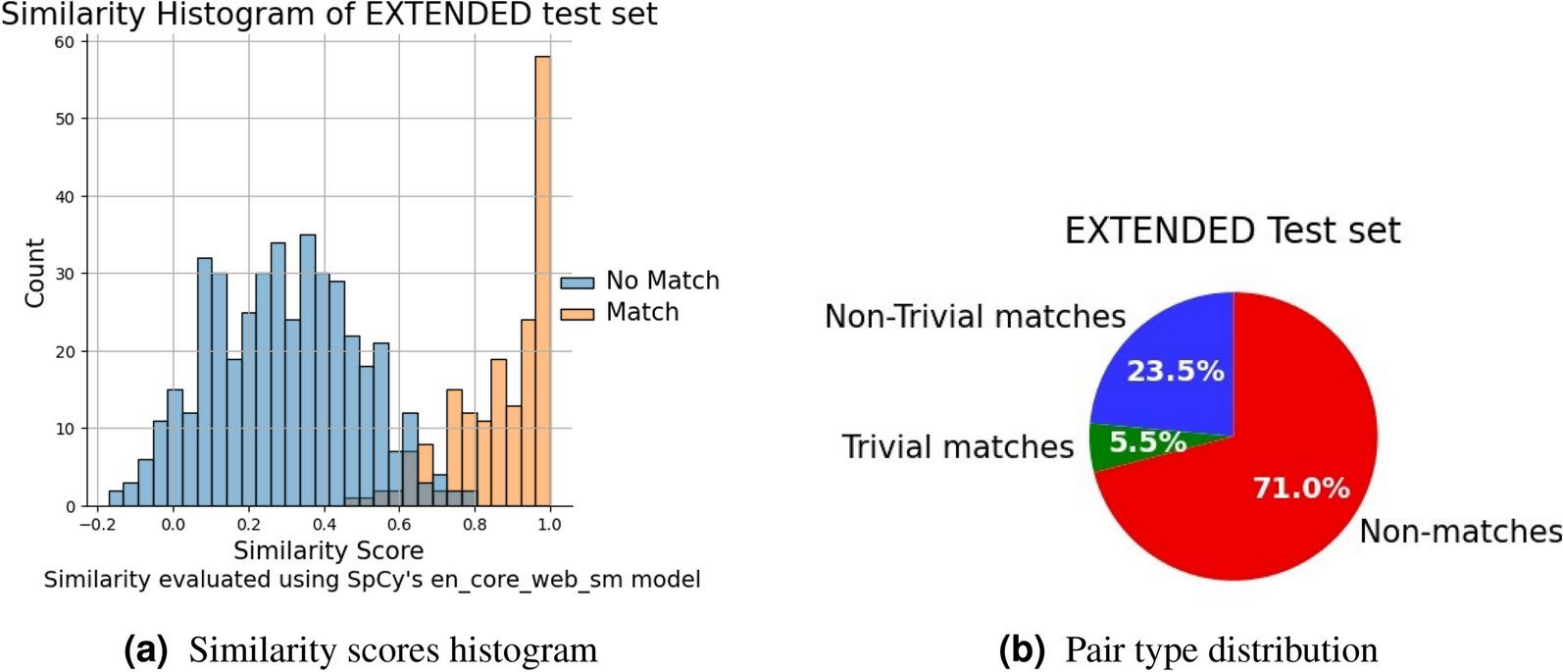
Visualization of the extended test set. The histogram shows on the x-axis the score of SpaCy’s similarity function, while on the y-axis the number of pairs. The pair type distribution shows the shares of the different pair types in the extended test set. It is noticeable that the number of matching pairs is increased.

To demonstrate the advantage of combining the HQNN model with TD-IDF, we first extended the existing test set to make it harder for the models to predict the correct label and to provide room for improvement, as both models already performed well on the original test set. The extended test set provides more matching pairs that are specifically in the region where the TF-IDF decision boundary is not clear. The test set extension now contains 603 rows. Details about the distribution of the similarity scores and the pair types in the extended test set are shown in Fig. 9.

**Fig. 10:**
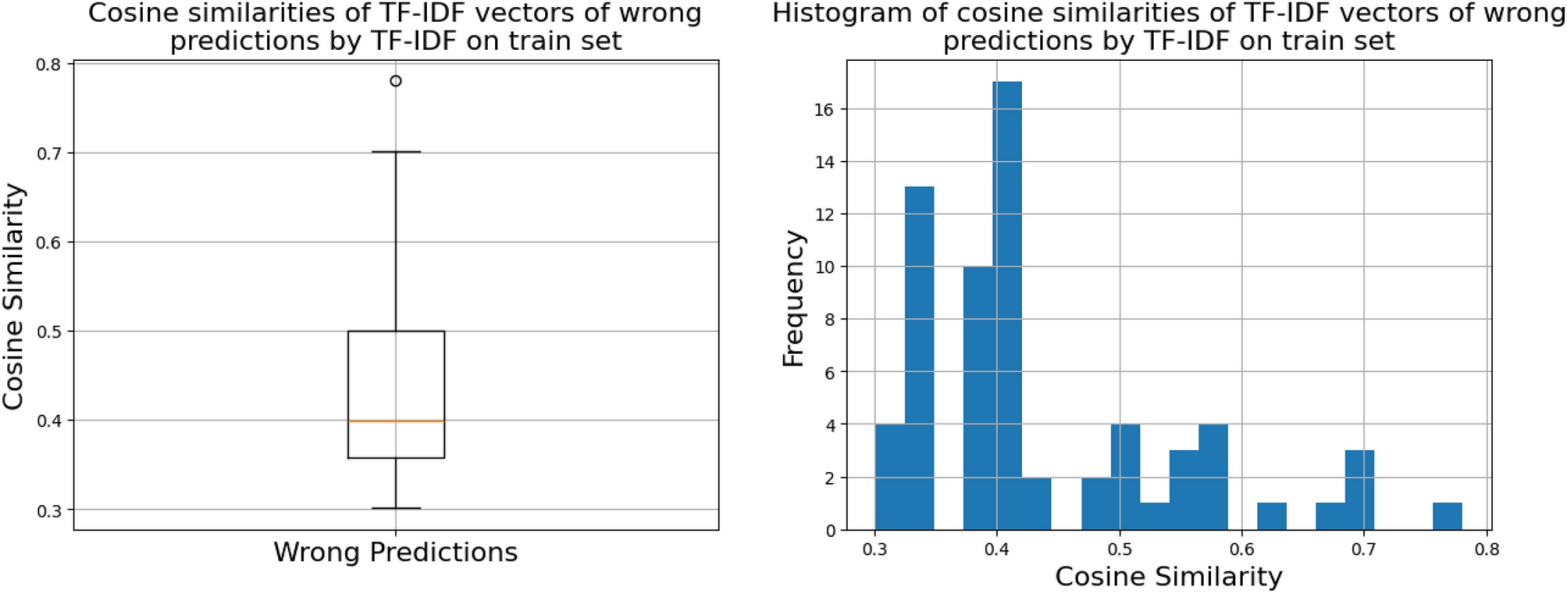
The TF-IDF model’s wrong predictions during training visualized by the TF-IDF similarity scores of the sentence pairs. The end of the first quartile is at 0.358, and the start of the third quartile is at 0.5. Note that unlike in the other similarity figures of this paper, the distribution is over the TF-IDF similarity scores.

Next, we had to find a reasonable threshold, for when the quantum model should be used. We did this by analyzing the TF-IDF similarity of the wrongly classified sentence pairs of the TF-IDF model during training, as shown in Fig. 10. Using the distribution of the similarity of the mistakes the TF-IDF model made, we can then calculate the first and third quartile. The region between the first and third quartile is where the quantum model should then be used since TF-IDF struggles to identify correct matches.

The results of the combination of the two models are shown in Table 4. It is noticeable that the performance of the extended data set is about 6% worse on average than on the original test set due to the harder matching cases. Combining the models can compensate for this. The beauty of this approach is that filtering out the extreme cases turns out to be a straightforward but very effective approach to boost the performance of the HQNN. Another noticeable change is the evaluation of the HQNN model on the real quantum computer using the extended test set. As IBM Hanoi was deprecated, the model was evaluated on IBM Osaka instead. However, IBM Osaka has a much higher qubit count than Hanoi, which could have influenced the results, even though the model only used 6 of the available 127 qubits, which probably caused the drop in performance.

### Transferring results between quantum simulator and quantum computer

Our experiments showed that a model trained using a quantum simulator is portable and thus transferable to a real quantum computer. In classical computing, this would not be a surprise, since the classical hardware is quite well understood, fault-tolerant and the behavior is reasonably predicable. However, in quantum computing, where hardware rapidly changes and fault tolerance is one of the main issues, it is not obvious that quantum simulators can model the underlying hardware sufficiently well such that the result of a simulator can serve as an initial setting for a quantum neural network. Roughly said, the simulator accounts for generic properties as one would expect them from theory and delivers an initial guess for the parameter settings of a quantum neural network. The fine-tuning on real hardware refines this guess, implicitly considering the hardware-specific distribution of errors. The results presented indicate that this distribution of labor works efficiently, at least in the context under scrutiny.

**Fig. 11:**
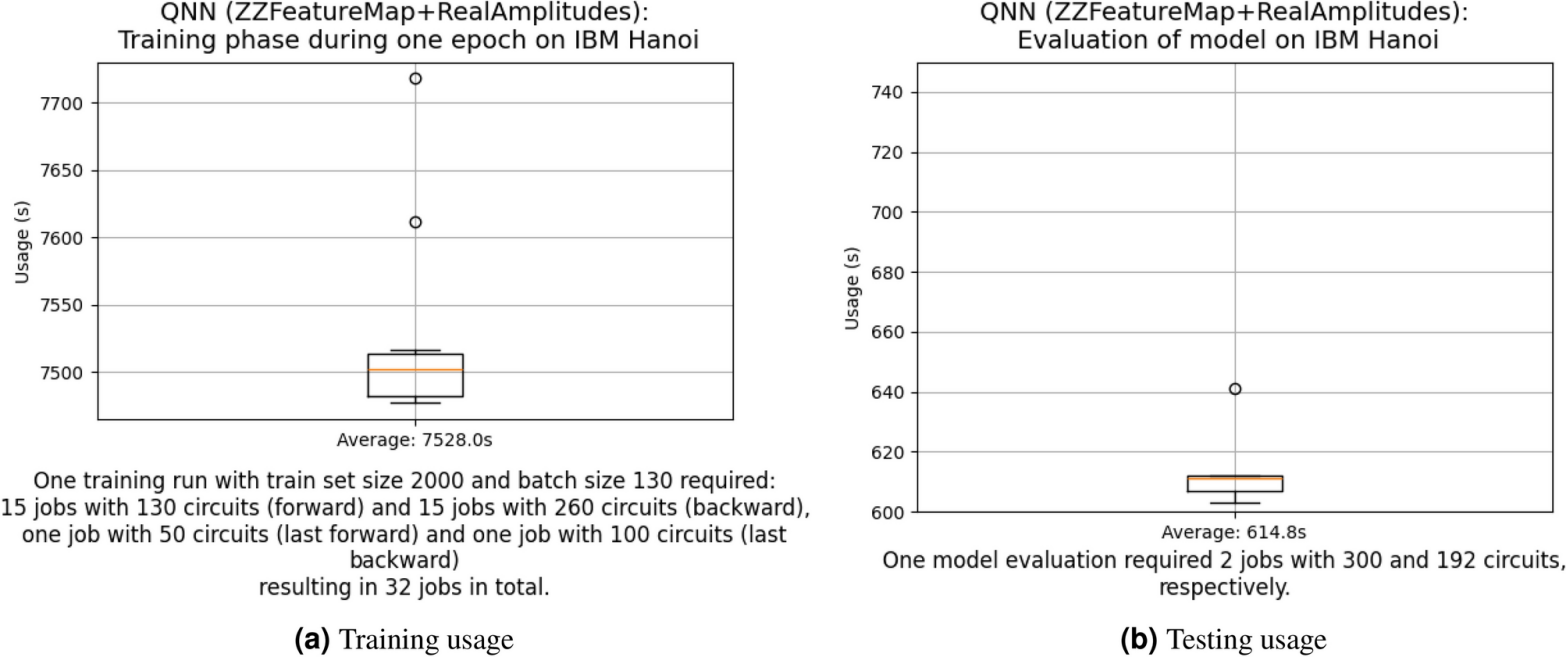
Usage of the IBM quantum computer during the training and evaluation of the QNN model.

Given the high costs associated with using real quantum devices, this is a crucial step towards practical applications and can save considerable amounts of money by pre-selecting the best models before moving them to the quantum computer. This already becomes visible when looking at the usage of the IBM quantum computer during the training of the QNN model, as shown in Fig. 11.

We have an average usage of 7528 s per epoch only for the training. Considering that we need at least 20 epochs for a good training, this results in 150,560 seconds or 41.8 h just spent on training, without validation or testing.

## Related work

Over the recent years, a vast variety of hybrid classical-quantum algorithms have been proposed, specifically in the field of quantum machine learning (QML). These typically involve a variational quantum circuit trained using some sort of classical optimization algorithm (e.g.^28,42–45^).

In the context of natural language processing (NLP), there has been work on a quantum approach of encoding meaning into a quantum state^27,46,47^, called the *distributional compositional model* (DisCoCat). This has also been proven to work in practice with smaller datasets (Lorenz et al.^48^) by performing sentence classification. The dataset size, however, is still limited by the current available quantum hardware. In their work, they used a small dataset with a simple context-free grammar, specifically aimed at testing the syntax sensitivity of the model.

Interesting for this paper is also the work by Ramesh and Vinay^49^, who implemented a quantum algorithm for string matching. Contrary to our work, they are aiming to find a substring in a larger string rather than a context-based comparison.

To the best of our knowledge, so far, no work has been done on the entity matching problem using quantum machine learning.

## Conclusion

In this paper, we evaluated a novel approach for entity matching using a combination of classical and quantum machine learning algorithms. We could not only demonstrate that a quantum layer can reach the performance of classical algorithms already in the NISQ-era but also by using far fewer parameters.

Our experimental results demonstrate that hybrid quantum neural networks (HQNN) work particularly well in cases where it is hard to distinguish between matches and non-matches. However, when dealing with trivial matches, HQNNs often struggle. To overcome this problem, we designed and evaluated a straightforward yet very effective approach of combining HQNN with TF-IDF as a filter. The basic idea is that the HQNN handles the matches that are hard to classify, while the TF-IDF tackles the cases that are easier to classify. Our combined approach shows the best performance over all other evaluated algorithms.

Our experiments also show that quantum machine learning models trained on quantum simulators are transferable to real quantum hardware, which can significantly reduce the costs of using expensive quantum devices. The basic idea is to do the major part of the training on a quantum simulator to reach reasonable parameter values and then fine-tune the trained model on a quantum device.

One of the major limitations of our approach is that it is applied on a relatively small dataset due to limits of current quantum hardware. However, in principle our approach is scalable to larger documents or even the more complex problem of multi-document matching^50^. The current limitations are the number of qubits that can be used for representing words. In order to enable matching of a larger number of words, a promising avenue of research is to use dimensionality reduction algorithms or quantum kernels to map a large problem space to a smaller one.

Moreover, the results demonstrate the feasibility of applying quantum machine learning for certain practical natural language processing problems even today. We expect that due to the rapid progress in quantum hardware and quantum algorithms, we will be able to tackle even larger and more realistic problems in natural language processing using quantum neural networks in the near future.

## Data Availability

Data sets generated during the current study are available from the corresponding author on reasonable request.
